# An observational cohort study to produce and evaluate an improved tool to screen older women with back pain for osteoporotic vertebral fractures (Vfrac): study protocol

**DOI:** 10.1007/s11657-019-0558-5

**Published:** 2019-01-25

**Authors:** T. K. Khera, A. Burston, S. Davis, S. Drew, R. Gooberman-Hill, Z. Paskins, T. J. Peters, J. H. Tobias, E. M. Clark

**Affiliations:** 1 Bristol Medical School, Faculty of Health Sciences, University of Bristol, Bristol, UK; 2 Health Economics and Decision Science, University of Sheffield, Sheffield, UK; 3 Arthritis Research UK Primary Care Centre, Research Institute for Primary Care and Health Sciences, Keele University, Staffordshire, ST5 5BG UK; 4 Haywood Academic Rheumatology Centre, Midlands Partnership NHS Foundation Trust, Stoke-on-Trent, ST6 7AG UK

**Keywords:** Vertebral fracture, Screening, Osteoporosis, Protocol

## Abstract

**Summary:**

The aim of this study is to produce an easy to use checklist for general practitioners to complete whenever a woman aged over 65 years with back pain seeks healthcare. This checklist will produce a binary output to determine if the patient should have a radiograph to diagnose vertebral fracture.

**Purpose:**

People with osteoporotic vertebral fractures are important to be identified as they are at relatively high risk of further fractures. Despite this, less than a third of people with osteoporotic vertebral fractures come to clinical attention due to various reasons including lack of clear triggers to identify who should have diagnostic spinal radiographs. This study aims to produce and evaluate a novel screening tool (Vfrac) for use in older women presenting with back pain in primary care based on clinical triggers and predictors identified previously. This tool will generate a binary output to determine if a radiograph is required.

**Methods:**

The Vfrac study is a two-site, pragmatic, observational cohort study recruiting 1633 women aged over 65 years with self-reported back pain. Participants will be recruited from primary care in two sites. The Vfrac study will use data from two self-completed questionnaires, a simple physical examination, a lateral thoracic and lateral lumbar radiograph and information contained in medical records.

**Results:**

The primary objective is to develop an easy-to-use clinical screening tool for identifying older women who are likely to have vertebral fractures.

**Conclusions:**

This article describes the protocol of the Vfrac study; ISRCTN16550671.

## Background and rationale

Osteoporosis is estimated to affect 200 million women worldwide [1]. Osteoporotic fractures are strongly associated with morbidity, especially in terms of pain and disability: out of the people who have had a fracture, 42% have chronic pain and 33% of these describe the pain as severe or unbearable [2]. People who are hospitalised after a vertebral fracture (VF) have a higher mortality rate following fracture than those hospitalised after a hip fracture [3]. Unlike mortality following a hip fracture, mortality after a VF rises progressively after the event [3]. However, unlike hip fractures, most VFs do not require hospitalisation. Furthermore, the socioeconomic burden of VF is not as well defined as that of hip fractures, in part because the epidemiology of VFs is less well established as there is no universally accepted definition.

Only 25% of VFs result from falls, with the majority caused by daily activities such as bending forwards, climbing stairs or lifting objects [4]. People with VFs have a reduced health-related quality of life that is not solely a result of confounding by age, nor can it be fully explained by the presence of pain [5]. VF in older people has an effect on everyday activities such as reducing their ability to get in and out of a car, lift objects from the floor, walk a few blocks, climb up and down steps, reach or extend their arms above shoulder level, prepare meals and do the shopping [6]. VFs can also deprive people of their social support because their physical and functional capabilities have been limited [7]. The presence of VFs increases the risk of subsequent fractures, especially further vertebral or hip fractures [8], although there are medications available to reduce this risk [9–11]. Despite this, less than a third of people with osteoporotic VFs come to clinical attention [12] due to a variety of reasons including lack of clear clinical triggers to identify who should have diagnostic spinal radiographs.

We previously carried out a cross-sectional study of 509 older women from primary care, identifying four clinical triggers that could be combined in a simple tool to determine who should have spinal radiographs to identify undiagnosed osteoporotic VFs: height loss, back pain, previous fracture as an adult and rib-to-pelvis distance [13]. A cutoff from the regression equation was subsequently selected which identified all women with more than one VF and half of those with one VF. A large randomised controlled trial (RCT) of this screening tool, Cohort for skeletal health in Bristol and Avon (COSHIBA), was then undertaken in a primary care population of unselected women aged over 65 years [14]; 3200 women were recruited. Allocation to the screening arm approximately doubled the prescription of osteoporosis medications at 6 months follow-up. However, preliminary cost-effectiveness modelling suggested a cost-per-QALY of £30,000, making it unlikely that this would be cost-effective from the National Health Service (NHS) perspective in this setting, in the UK. This was mainly because of a low prevalence of VFs in this unselected population. A key finding from the National Osteoporosis Society into the realities of life with osteoporosis was that 58% of people with VFs have long-term back pain [15]. Therefore, we carried out a case-control study to look more specifically at the population of women aged over 60 years with back pain [16] to identify self-reported characteristics of pain that might help predict who has osteoporotic VFs. This study identified novel independent predictors of VF including shorter duration of back pain, pain described as crushing, pain improving on lying down and pain not spreading down the legs. Area under the curve (AUC) statistics for the combination of these factors to identify women with VFs was 0.85 (95%CI 0.79 to 0.92). Therefore, self-reported pain descriptives may identify people with back pain who have a vertebral fracture [16–18]. The Vfrac study combines the original COSHIBA screening tool with the newly identified pain descriptors (McGill questionnaire and new qualitative focus group work). Only women over 65 years with self-reported back pain will be eligible for this study. The aim of Vfrac is to produce and evaluate an improved screening tool (Vfrac) for use in older people presenting to primary care with back pain.

### The need for this study

The National Institute for Health and Care Excellence (NICE) guidelines CG146 [19] state the requirement for a study based in primary care to assess which people are at high risk of fracture. The Arthritis Research UK metabolic bone disease musculoskeletal trauma clinical studies group strategy suggested studies of new methods of detection of vertebral fractures and their effects on quality of life as one of their five focuses [20]. Furthermore, patients with osteoporosis and fracture identified earlier identification of osteoporosis as a top 2 research priority in a UK study of 1188 patients [21].

If Vfrac is deemed to be cost-effective, national implementation of Vfrac in primary care and local Fracture Liaison Services is likely to identify more of the currently undiagnosed older women with osteoporotic VFs, who have one of the highest risks of future fracture [22, 23], and will allow reduction in future fracture risk by approximately 50% after intervention [24].

### Primary aim

The aim of this Arthritis Research UK funded study (ref: 21507) is to produce and evaluate an improved tool (Vfrac) to screen older women with back pain for osteoporotic vertebral fractures.

## Design and methods

### Outline

The Vfrac study is a multicentre, pragmatic, observational cohort study with data collection at baseline and 3 months follow-up. Baseline data collection is by self-report, physical examination at a research clinic plus spinal radiographs. Data collection at follow-up is by self-report and from GP electronic records. Table 1 shows the timeline for the study.

**Table 1:** Vfrac study timeline

	2017	2018				2019				2020				2021	
	Pre-funding	Jan–Mar	Apr–Jun	Jul–Sep	Oct–Dec	Jan–Mar	Apr–Jun	Jul–Sep	Oct–Dec	Jan–Mar	Apr–Jun	Jul–Sep	Oct–Dec	Jan–Mar	Apr–Jun
Ethics, research governance and other approvals; staff recruitment	●	●	●												
Recruitment of general practices			●		●		●								
Recruitment of patients				●	●	●	●	●	●	●					
Data collection: self-report (baseline questionnaire) and physical examination				●	●	●	●	●	●						
Spinal radiographs				●	●	●	●	●	●	●					
Statistical analysis												●	●		
Generate web-based Vfrac tool													●	●	
Identification of stopping rules for future definitive trial													●	●	
Follow-up of patients having X-rays (follow-up questionnaire and medical records download)					●	●	●	●	●	●	●				
Modelling cost-effectiveness													●	●	●
Dissemination, and preparation of future pilot application			●								●		●	●	●

**Fig. 1:**
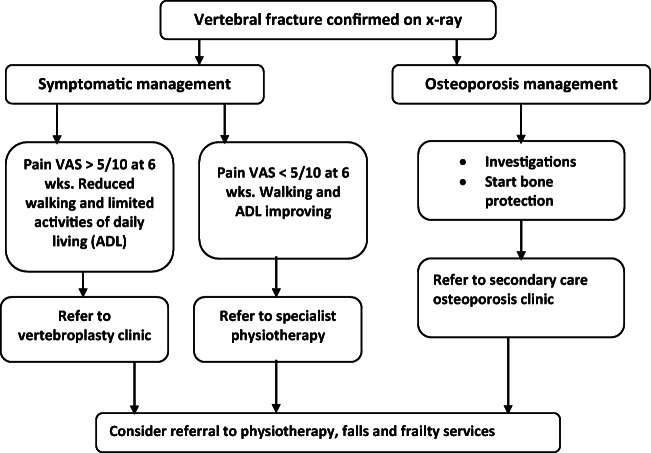
Management of a patient with an osteoporotic vertebral fracture. Note: (Manage patients according to this pathway provided they have no recent history of significant back trauma, no red-flag clinical features to suggest alternative diagnoses such as malignancy and are at risk of osteoporosis (e.g. FRAX score amber or red—see http://www.shef.ac.uk/FRAX/tool.jsp)

A total of 1633 women aged over 65 years will be recruited via primary care from two sites within England — Bristol and Stoke-on-Trent. Potential participants will be asked for their consent via post and asked to fill in a baseline questionnaire containing items on demographics, socioeconomic status, traditional risk factors for osteoporosis, back pain, quality of life, medication use, healthcare utilisation and comorbidity. Upon the study team’s receipt of the completed baseline questionnaire and consent form, an appointment will be made for the participant to have a physical examination in secondary care followed by a lateral thoracic and lateral lumbar radiographs performed using standard NHS standard operating procedure on the same day. Before the physical examination, the participant will be asked, in person, to sign another consent form. In this consent process, they will be asked if they agree to the physical examination and radiograph. The radiograph report will be sent to the participant’s general practitioner (GP) as if the GP had ordered the radiograph. The GP practices will be given a guidance document upon recruitment, providing information on recommended pathway for patients with a vertebral fracture. This advice has been taken from the North Bristol NHS Trust guidance for GP practices within BSSG CCG (Fig. 1). The first step in this guidance is an X-ray to confirm VF.

**Fig. 2:**
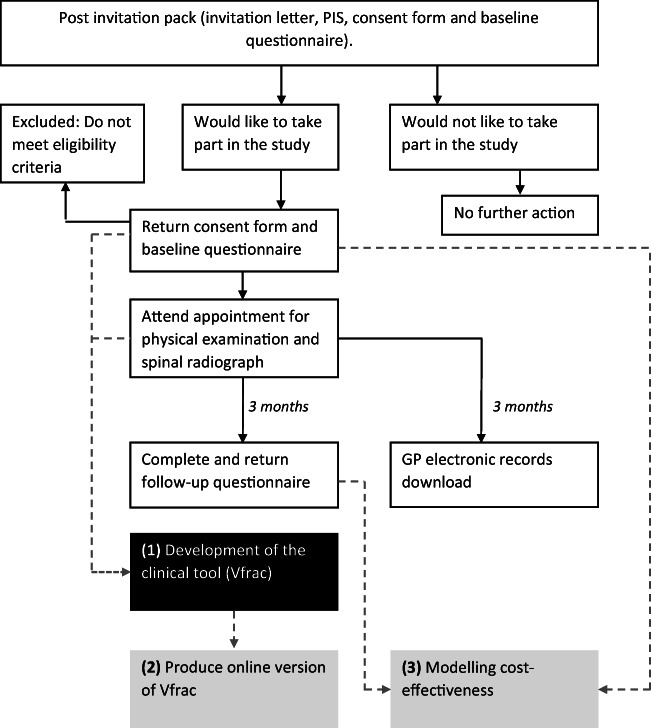
Study flowchart

A copy of the radiograph and the report will also be sent to the chief investigator for research analysis after identifying information has been removed. Three months after the radiograph, the participant will be asked to complete a follow-up questionnaire, containing questions about whether results of the spinal radiograph were communicated to the patient, whether they started on any new medication, and whether they have been referred to any healthcare service, e.g. physiotherapy and quality of life. Data collection from the participants’ GP electronic records will also be conducted at this time. This will comprise extraction of information about current medication, number of contacts with healthcare practitioners, whether results of the radiograph are recorded in the notes and concomitant illnesses. The end of study will be the date of receipt of the last follow-up questionnaire of the last patient. The study flowchart is provided in Fig. 2.

The primary objective is to develop a clinical screening tool for identifying older women with back pain who are likely to have VF. The output will be a simple clinical checklist of self-reported data and data from the physical examination which will produce a binary assessment of (1) yes—this person needs a spinal radiograph, or (2) no radiograph needed. The primary evaluative measure will be the accuracy of the Vfrac tool as represented by the sensitivity and specificity of > 80%.

### Study participant selection

General practices will be recruited from Bristol (9) and Stoke-on-Trent (16) areas. Practices will be recruited after an open call in the order they express an interest in the study until the number of participants target is reached at each site. The following entry criteria will be used for participants:

Inclusion: participant is willing and able to give informed consent for participation in the study, female, over 65 years and with self-reported back pain (via baseline questionnaire) in the previous 4 months.

Exclusion: male, 65 years or under, no self-reported back pain (via baseline questionnaire) in the previous 4 months, has already had a full spinal X-ray in the previous 4 months or considered unsuitable to take part by their GP (e.g. cognitive impairment, near end of life or recently bereaved).

### Sample size

The sample size is based on the following assumptions:

- A prevalence of VFs between 12 and 20%, based on data from the European Vertebral Osteoporosis Study (EVOS) [12]. This is based on the general population of women under 65 years and is, therefore, likely to be lower than the true prevalence of VFs in older women with back pain.
- A margin of error (that is, the half-width in 95% confidence intervals of sensitivity or specificity) of 5%.
- Sensitivity and specificity of the Vfrac tool between 80 and 95%.

Table 2 shows the range of sample sizes according to prevalence of VFs and sensitivity or specificity of the Vfrac tool; we will aim for a sample size of 1633. This sample size will be large enough to encompass any specificity of Vfrac as sample sizes required for specificity are much lower.

**Table 2:** Calculation showing range of sample sizes according to prevalence of vertebral fractures and sensitivity (A) or specificity (B) of the Vfrac tool

	Prevalence of vertebral fractures		
	12%	15%	20%
Sensitivity			
80%	2050	1640	1230
85%	1633	1306	980
90%	1159	927	695
95%	609	487	365
Specificity			
280	289	308	80%
232	231	245	85%
158	164	174	90%
83	86	92	95%

### Recruitment

Recruitment will be from the community via primary care. Women over 65 years who can give informed consent and are not found to be unsuitable for the study by their GP (e.g. are not housebound) will be sent study invitation packs. The invitation pack will contain an invitation letter signed by the potential participant’s GP, the participant information sheet, two copies of the first consent form and the baseline questionnaire. The invitation packs will be sent to the potential participants by their own general practitioner. The invitation pack will make it clear that the study wishes to include women over 65 years who have had back pain in the previous 4 months, even if they have had back pain for more than 4 months. Reminder letters will be sent after 4–6 weeks if no response is initially received.

Participants willing to participate will be asked to complete the baseline questionnaire and one copy of the consent form, returning these to the researchers using a prepaid envelope. The consent form will contain a section for the participant to supply us with their title, name, address, phone number, email address and contact preferences.

Upon receipt, the completed consent form and baseline questionnaire will be checked by a study researcher. The first question in the baseline questionnaire is a screening question: ‘have you had back pain in the previous 4 months?’. If the participant has answered ‘no’, then they are not eligible for the study and will be sent a thank you letter. If a potential participant is eligible, they will be booked in for a physical examination and spinal radiographs. A research administrator will phone the participant to make the appointment after ensuring the participant is willing to continue taking part in the study and has not had a full spinal radiograph in the previous 4 months. Participants will be asked to give written face-to-face consent for the physical examination and the radiographs at the research clinic.

Each participant has the right to withdraw from the study at any time. If any participant loses capacity during the study, they will be withdrawn but their data to date kept.

### Follow-up

Follow-up will occur 3 months after the spinal radiograph via post and through data collection from GP electronic records. The participants will receive a follow-up questionnaire and cover letter. If there is no reply after 2–3 weeks, the participant will receive another follow-up questionnaire and reminder cover letter.

## Data collection and analysis

Data collection will occur at baseline via self-report, a physical examination at a research clinic and spinal radiograph, and at follow-up via self-report and through GP electronic records.

The items in the questionnaires were chosen based on literature review and previously published work [13, 14, 16]. The questionnaires will contain the following items:

- Margolis pain diagrams [25] at baseline
- PHQ-9 [26] at baseline and follow-up
- PainDETECT [27] at baseline
- FRAX [28] clinical risk factors only at baseline
- Q-Fracture [29] at baseline
- Diagnosis of osteoporosis at baseline and follow-up (‘have you ever been diagnosed with osteoporosis (brittle bones)?’; ‘approximate date of diagnosis’)
- Traditional risk factors of osteoporosis at baseline
- EQ5D-5L [30] at baseline and follow-up
- ICECAP-O [31] at baseline and follow-up
- Items based on focus group work with patients with VFs and based on the McGill Pain questionnaire [16, 18] at baseline
- Descriptions of pain deemed important during focus group work (REC reference number16/NS/0110) at baseline
- Use of pain medication at baseline and follow-up
- Current prescription drugs and over the counter medication/remedies/supplements at baseline and follow-up
- Use of healthcare services at baseline and follow-up
- Whether results of the radiograph were communicated to the patient at follow-up

During the research clinic, the following information will be collected: height (stand-alone stadiometer), weight, chest expansion, rib-to-pelvis distance, wall-tragus distance and waist circumference. To calculate height loss, we will be relying on the participant to know how tall they were at 25 years. In the baseline questionnaire, the participants are asked ‘how tall were you at aged 25?’ and ‘how tall are you now?’. These data were chosen based on literature review and previous published research [13, 14, 16]. The Vfrac study chief investigator will have access to all spinal radiographs with the study ID number as the only identifying feature.

### Objectives and outcome measures

(1) To develop a clinical screening tool for identifying older women with back pain who are likely to have VFs. The output of this aim is a simple clinical checklist likely to consist of self-reported data and data from physical examination. The primary evaluative measure will be the accuracy of the Vfrac tool as represented by the sensitivity and specificity. The aim is for sensitivity and specificity of > 80% to justify subsequent evaluation in a definitive trial. Data from the baseline questionnaire, the physical examination and spinal radiograph will be used.

(2) To produce an online version of the combined predictors for easy use as a clinical tool (Vfrac) to identify which older women with back pain are likely to have a VF and, therefore, needs a radiograph. The output of this aim is a web-based online version of the clinical checklist (see primary objective).

(3) To model the cost-effectiveness of Vfrac based on follow-up of the participants to identify if a future definitive cluster randomised controlled trial is appropriate. The output of this aim will be an estimate of likely cost-effectiveness and a list of sources of variation that can be tested in a pilot as part of a future application for funding. Data at follow-up by self-report plus from GP electronic records will be used.

### Analysis of outcome measures—vertebral fractures

All spinal radiographs will be analysed by a trained clinician researcher for the presence of VFs using the Algorithm-Based Qualitative (ABQ) approach [32]. Images that are difficult to interpret via the ABQ method or using automated QM will be viewed by the trained clinician researcher (EC) and a spinal radiologist and consensus reached. In ABQ, the diagnosis of osteoporotic fracture assumes that these fractures always involve the endplate within the vertebral ring. This method recognises that short vertebral height is not always due to fracture [33], but may be developmental or degenerative in origin. As such, the ABQ is felt to be more specific for true VF, particularly mild deformities and avoids the high false-positive rate of quantitative morphometry (QM) [34]. In addition, QM [35] will also be used to identify VFs as this method is available within automated software for the analysis of spinal radiographs and has the potential to become more commonly used within the NHS. However, QM is less specific and is associated with an increased number of false positives.

### Analysis—developing the clinical tool

Data from the questionnaires and physical examination will be linked to those from the analysis of spinal radiographs for the presence/absence of vertebral fractures. The first step will be univariable analyses to identify associations between risk factors and presence of VFs. Multiple logistic regression analyses will be performed with the aim of identifying clinical risk factors that are independently predictive of the presence of VF. All likely contenders from the univariable analysis will be included, with variables deleted in a backwards fashion whilst considering aspects such as clinical interpretation and associations between the variables, rather than relying on a simple automated basis. For example, once a final model is obtained, all deleted variables will be checked one by one to confirm if they did not contribute further. Hence, the number of variables to be considered in the multivariable modelling has not been pre-specified (on, for instance, an events per variable threshold), but the analyses will be structured, including considering variables in cognate groups, so that it is unlikely that any one model will involve more than 6–10 variables [36]. Separate analyses will then be performed to identify a cutoff to predict one or more VFs and more than one VF. Sensitivity and specificity of the pre-determined threshold will be calculated using standard techniques, comparing against the gold standard of identification of VFs from radiographs by the chief investigator using the ABQ method.

Secondary analyses will involve calculating the AUC to measure discriminative ability. To provide an estimate of the validity of the predictive models, 200 bootstrap samples of the original dataset will be derived (each bootstrap sample will be generated by randomly sampling from the original dataset with replacement 505 times). Logistic regression will then be performed on each bootstrap sample using the same set of predictor variables as in the final model, and the regression coefficients and AUC calculated. Each set of bootstrap sample coefficients will then be used to calculate AUC for the original dataset. The difference between these estimates will be used to evaluate the extent to which the discriminative ability of our final model decreases when applied to other random samples [37].

### Analysis—modelling cost-effectiveness

Ideally, the presence of an osteoporotic VF will result in formal assessment of future fracture risk and prescription of medications such as bisphosphonates. Follow-up data will allow identification of patient pathways after patients have received their spinal radiograph. This will allow estimation of the number of older women receiving new bisphosphonate (or other anti-osteoporosis medications) prescriptions and whether they had any further investigations (such as a DEXA scan) or additional primary care contacts, including physiotherapy after their radiograph. A modelling approach will be used to estimate the cost-per-quality adjusted life year (QALY) if Vfrac is implemented in the NHS. The EQ-5D data collected during this study will provide a baseline QALY for older women with back pain in the UK, as these data are not currently available. Data from the literature will be used to model the likely difference in change in QALYs between any future control and intervention arms. These will be based upon estimated changes in QALYs including, for example, due to age, occurrence of osteoporotic fracture, management of back pain and prescription of medications for osteoporosis.

Without a randomised element, it is not possible to measure the proportion of women going on to get the correct diagnosis of VF and appropriate treatment in the absence of the Vfrac tool. It is, therefore, necessary to explore alternative scenarios using expert opinion. Opinion of experts in the fields of primary care, osteoporosis and care of the elderly will be obtained on likely patient pathways in the absence of Vfrac. Interviews and group discussions will be undertaken with physicians, surgeons, GPs and allied health professionals in the fields of primary care, osteoporosis and care of the elderly over a 6-month period will provide an estimation of each likely step in the treatment pathway of women with back pain occurring in the absence of Vfrac including to estimate costs of any adverse effects of medications.

As individual-level data will be available, the uncertainty (variance) of our cost-effectiveness analysis will be estimated via a probabilistic uncertainty approach using Monte Carlo Simulations in combination with non-parametric bootstrapping techniques, where appropriate. The quality and validity of the cost-effectiveness model will be ensured by (1) basing the structure of the model on previous cost-effectiveness models in the field of osteoporosis; (2) ensuring inputs to the model are valid; (3) ensuring modelling is appropriated to the future decision-making context of Vfrac (that is, from the NHS perspective and tailored to CCGs); and (4) the uncertainty (variance) is clearly identified so it can be tested in any future pilot study.

## Access to data

Direct access will be granted to authorised representatives from the sponsor and host institution for monitoring and/or audit of the study to ensure compliance with regulations. Patients will be asked for permission to share anonymised data beyond the immediate project team. The data will be deposited at the University of Bristol Research Data Repository (as controlled data). A metadata record will be published openly by the repository and this record will clearly state how data can be accessed.

## Quality assurance procedures

The study will be monitored or audited in accordance with the currently approved protocol, good clinical practice, relevant regulations and standard operating procedures of the sponsor. A Vfrac study Steering Committee, with terms of reference as per the funder’s guidelines, and will meet twice per year between 1st January 2018 and 30th June 2021. The. The Vfrac management team will meet monthly.

## Ethical and regulatory considerations

The investigator will ensure that this study is conducted in accordance with the principles of the Declaration of Helsinki. The chief investigator will ensure that this study is conducted in accordance with relevant regulations and with good clinical practice.

## Publication policy

Upon study completion, a report will be prepared for the funding body. The results will be published in peer-reviewed journals and presented at scientific meetings. Arthritis Research UK and the University of Bristol’s open access policies for publication of peer-reviewed papers will be followed.

The chief investigator and co-applicants will be involved in reviewing drafts of the manuscripts, abstracts, press releases and any other publications arising from the study. Authors will acknowledge that the study was funded by Arthritis Research UK. Authorship will be determined in accordance with the ICMJE guidelines and other contributors will be acknowledged.

A poster summarising the results will be sent to all GPs involved with the study to display in their waiting area. Any patients who give us their email address will be sent a copy of this poster. A summary of the results will also be posted on the study webpage: http://www.bristol.ac.uk/translational-health-sciences/research/musculoskeletal/rheumatology/research/vfrac-study.html

## Conclusion

If successful, this planned cohort study will result in a simple checklist ready for testing within the NHS to identify which older women with back pain should have a spinal radiograph as they are at high risk of having a VF. Correct identification of women with VFs will allow them to have medications and other treatments to reduce their risk of future fracture by half [24]. This is likely to have substantial impacts on both NHS healthcare costs (by reducing hip fracture costs, for example) as well as improving quality of life for older people.
